# Plasma GDF15 levels associated with circulating immune cells predict the efficacy of PD-1/PD-L1 inhibitor treatment and prognosis in patients with advanced non-small cell lung cancer

**DOI:** 10.1007/s00432-022-04500-5

**Published:** 2022-12-06

**Authors:** Green Hong, Pureum Sun, Chaeuk Chung, Dongil Park, Song-I Lee, Nayoung Kim, Seong Eun Lee, Jeong Eun Lee, Yea Eun Kang, Da Hyun Kang

**Affiliations:** 1 Department of Internal Medicine, College of Medicine, Chungnam National University, Daejeon, Korea; 2 Institute for Medical Sciences, College of Medicine, Chungnam National University, Daejeon, Korea

**Keywords:** GDF15, Non-small cell lung cancer, Immunotherapy, Survival, Biomarker, Efficacy

## Abstract

**Purpose:**

Although increased plasma growth differentiation factor-15 (GDF15) levels have been reported in patients with various cancers, the predictive role of PD-1/PD-L1 inhibitors in advanced cancers remains unknown. This study aimed to investigate GDF15 levels as a predictive marker in advanced non-small cell lung cancer (NSCLC) treated with PD-1/PD-L1 inhibitors and analyze their association with immune cell populations.

**Methods:**

This study included 87 patients with advanced NSCLC receiving anti-PD-1/PD-L1 inhibitors between March 2018 and May 2020. Blood samples were obtained immediately before and months after PD-1/PD-L1 inhibitor administration.

**Results:**

The objective response rate (ORR) was significantly higher in the low GDF15 than in the high GDF15 group (39.2% vs. 15.3%, *P* = 0.013). The median progression-free survival (PFS) was significantly longer in the low GDF15 than in the high GDF15 group (13.2 [95% CI 7.6–18.9] vs. 7.2 [95% CI 4.8–9.6] months, *P* = 0.048). Moreover, plasma GDF15 levels negatively correlated with PD-1^+^/CD8^+^ T cells (*r* = − 0.399, *P* = 0.003) and positively with PD-1^+^/Treg cells (*r* = 0.507, *P* < 0.001) and PD-1^+^Treg/CD4^+^ T cells (*r* = 0.439, *P* < 0.001). The ORR was significantly higher in the group with decreased GDF15 from baseline than in the increased GDF15 group (37.2% vs. 10.0%, *P* = 0.026). The median PFS was significantly longer in the decreased GDF15 group (14.8 [95% CI 10.4–19.2] vs. 5.9 [95% CI 2.8–9.0] months, *P* = 0.002). Plasma GDF15 levels were associated with PD-1^+^CD8^+^ T cells and PD-1^+^ Treg cells.

**Conclusion:**

Plasma GDF15 could be a potential biomarker for predicting the efficacy and survival benefit of immunotherapy in advanced NSCLC.

**Supplementary Information:**

The online version contains supplementary material available at 10.1007/s00432-022-04500-5.

## Introduction

The survival of patients with advanced non-small cell lung cancer (NSCLC) has recently improved with the use of new chemotherapeutic agents, such as targeted agents and immune checkpoint inhibitors (ICIs); these include nivolumab, pembrolizumab, and atezolizumab (Anagnostou and Brahmer 2015; Doroshow et al. 2019). ICIs have shown favorable antitumor effects and treatment duration compared with cytotoxic chemotherapy (Horn et al. 2017; Herbst et al. 2016). Although PD-L1 expression in tumors is the only biomarker used in patients treated with ICIs, it has low predictive power (Patel and Kurzrock 2015). Numerous studies on various biomarkers, including tumor mutation burden, have been conducted, but there are still few clinically useful ones (Sacher and Gandhi 2016; Sholl 2022; Yarchoan et al. 2019). Recently, the combination of PD-1/PD-L1 inhibitor and various drugs, including other ICIs, CTLA inhibitors, and anti-angiogenetic agents, is gradually becoming the standard treatment due to primary and acquired resistance to ICIs (Passiglia et al. 2021; Vafaei et al. 2022). Some patients do not initially respond to ICI, and although some patients respond well, resistance develops over time (Bai et al. 2020). In other words, it is very important to discover a new target that can not only predict response but also overcome resistance to ICI.

Growth differentiation factor 15 (GDF15) is a stress-induced cytokine known as a divergent member of the transforming growth factor-β (TGF-β) superfamily (Luan et al. 2019). Recently, GDF15 was shown to be induced in many cell types under conditions of stress, such as metabolic disease (diabetes and obesity), inflammation, infection, cardiovascular disease, and cancer (Chung et al. 2017; Kang et al. 2021; Johann et al. 2021). Recent evidence has shown that GDF15 causes anorexia and weight loss by binding to its receptor GDNF family receptor alpha-like (GFRAL), which is mainly expressed in the hindbrain and enables signaling through proto-oncogenic signaling. However, the mechanism of GDF15 in peripheral tissues is not fully understood (Mullican et al. 2017; Rochette et al. 2020; Yang et al. 2017). Although elevated circulating GDF15 levels in patients with cancer have been frequently reported (Wang et al. 2017; Suzuki et al. 2021; Song et al. 2021), the conflicting effect of this cytokine in predicting disease progression or response to chemotherapeutic agents in cancer has not been fully elucidated. One previous study reported that higher serum GDF15 levels were an independent risk factor for reduced overall survival (OS) in patients with early NSCLC (Liu et al. 2016). However, the predictive role of GDF15 in patients with advanced NSCLC who underwent immunotherapy is also not fully understood.

Recently, an emerging role for GDF15 in immune cell phenotypes of various diseases has been identified (Wischhusen et al. 2020a, b). The depletion of tumor-derived GDF15—directly regulated by NF-κB—in an orthotopic pancreatic cancer model restored the immune surveillance function of tumor-infiltrating macrophages, resulting in improved tumor control (Ratnam et al. 2017). GDF15 is known to promote immune escape of tumor cells by inhibiting T cell stimulation and cytotoxic T lymphocyte activation (Haake et al. 2022). Although it has been reported that GDF15 expression in human tumor tissues is associated with CD3+ or CD8+ T cell infiltration (Wischhusen et al. 2020a, b), the effect of circulating GDF15 on immune escape signaling remains largely unknown. In the present study, we investigated whether GDF15 levels can be a predictive marker in patients with NSCLC treated with PD-1/PD-L1 inhibitors and identified the association between circulating GDF15 levels and immune cell populations of peripheral blood mononuclear cells (PBMCs).

## Materials and methods

### Patients and treatment

This was a prospective cohort study which included patients with NSCLC who were treated with PD-1/PD-L1 inhibitor monotherapy at Chungnam National University Hospital (CNUH) between March 2018 and May 2020. To exclude the influence of various acute systemic clinical factors affecting plasma GDF15 levels, the inclusion criteria were as follows: age > 18 years, no clinical signs of infection or inflammation, and no pregnancy.

Collected demographic characteristics included age, sex, smoking history, and the Eastern Cooperative Oncology Group performance score (ECOG PS). In addition, we evaluated cancer-related information, including histologic type of lung cancer, clinical stage, type of agents, number of prior regimens, and number of metastatic sites. Intravenous nivolumab (3 mg/kg of body weight every 2 weeks), pembrolizumab (2 mg/kg of body weight every 3 weeks in previously treated patients and 200 mg in previously untreated patients), or atezolizumab (1200 mg every 3 weeks) were administered to patients. Treatment was continued until the patient experienced serious adverse effects, had confirmed investigator-assessed disease progression, or withdrew from the study. Patients who were expected to experience clinical benefits could continue treatment beyond radiological disease progression.

Peripheral blood was collected from the patients before treatment (day 0) and at the first response evaluation after receiving PD-1/PD-L1 inhibitors. PBMCs were isolated from whole blood using standard Ficoll-Paque (GE Healthcare) density gradient centrifugation in patients available for pre-treatment blood samples.

This study was conducted in accordance with the Declaration of Helsinki and Good Clinical Practice guidelines and was approved by the institutional review board of CNUH (2018-01-059). All patients were required to provide written informed consent before participating in this study.

### PD-L1 expression

PD-L1 expression was assessed by qualitative immunohistochemical (IHC) staining using the in vitro diagnostic PD-L1 IHC 22C3 pharmDx test (Agilent Technologies, Santa Clara, CA, USA) on the Dako Autostainer (Dako, Carpinteria, CA, USA) and the PD-L1 IHC SP263 test on the Ventana BenchMark platform (Ventana Medical Systems, Tucson, AZ, USA). The percentage of immunoreactive tumor cells was quantified according to the manufacturer’s recommendations. Cancer cells were considered positive when cell membrane staining was present, ignoring pure cytoplasmic immunoreactions. Immune cell staining was disregarded. PD-L1 protein expression was determined based on the percentage of viable tumor cells showing partial or complete membrane staining (tumor proportion score, TPS) (Park et al. 2019). We designed three categories of PD-L1 expression according to TPS cutoffs of 1% and 50%: no (< 1%), low (1–49%), and high (≥ 50%) PD-L1 expression. The classification of subgroups according to PD-L1 expression was based on the results of the 22C3 pharmDx assay, and patients without the 22C3 pharmDx assay results were classified based on the SP263 assay.

### Plasma measurements of GDF15, soluble PD-1, and soluble PD-L1 levels

Blood was drawn in the morning after overnight fasting. Plasma was obtained within 2 h of venipuncture by centrifugation at 2000×*g* for 20 min at 4 °C, rapidly frozen, and stored at − 80 °C until analysis. Plasma GDF15 levels were measured using a quantitative sandwich enzyme-linked immunosorbent assay (ELISA) kit (R&D Systems, Minneapolis, MN, USA; Quantikine ELISA kit for human GDF15, catalog no. DGD150). Plasma soluble PD-L1 levels were measured using a human PD-L1 Quantikine ELISA kit (R&D Systems, catalog no. DB7H10). Plasma soluble PD-1 levels were measured using a human PD-1 DuoSet ELISA kit (catalog no. DY1086).

### Treatment response and survival analysis

A response assessment with computed tomography was performed every three cycles for patients treated with pembrolizumab or atezolizumab and every four cycles for patients treated with nivolumab. Response to ICI treatment was assessed based on the Response Evaluation Criteria in Solid Tumors version 1.1. Clinical benefit was defined as the disease control rate (DCR), including complete response, partial response, and stable disease.

Treatment duration of ICI was defined as the time from the date of the first ICI treatment to the date of the last treatment. Progression-free survival (PFS) was defined as the time from the date of the first ICI treatment to the date of documented progression or death from any cause. OS was measured from the date of the first ICI treatment to the date of death or the last day of follow-up.

### Multi-color flow cytometry

Multi-color flow cytometry of PBMCs was performed at CNUH. The following human antibodies were used for multi-color flow cytometry: Brilliant Violet 421-conjugated anti-CD25, PE-Cy7 conjugated anti-CD45RA, and APC-Cy7 conjugated anti-CD3 (BD Biosciences, San Jose, CA, USA), and Brilliant Violet 605-conjugated anti-CD8, PerCP-Cy5.5-conjugated anti-CD4, FITC-conjugated anti-PD-1, and APC-conjugated anti-CD127 (BioLegend, San Diego, CA, USA). For intracellular staining of FOXP3, cells were fixed and permeabilized after surface staining using the Foxp3/Transcription factor staining buffer set (eBioscience) and incubated with a PE-conjugated FOXP3 antibody (eBioscience, San Diego, CA, USA). To exclude dead cells, single-cell suspensions were first incubated for 20 min in a viability dye (LIVE/DEAD Fixable Aqua, Thermo Fisher). The stained cells were analyzed using BD LSR Fortessa X-20 flow cytometry (BD Biosciences). Fluorescence-activated cell sorting was performed using FlowJo software (Tree Star, Ashland, OR, USA).

### Statistical analysis

To calculate the sensitivity and specificity of the biomarkers, conventional receiver-operating characteristic (ROC) curves were generated, and the area under the curve (AUC) was calculated. The optimal cutoff value was determined as the point at which the Youden index was maximized by the ROC curve. All data are expressed as mean ± standard deviation. Chi-squared and independent *t* tests were used to analyze differences in the patients’ clinicopathological data. Multivariate analysis was performed using logistic regression analyses. Furthermore, Pearson’s correlation coefficients were calculated to analyze the relationships among the variables. Survival was estimated using the Kaplan–Meier method, and survival rates were compared using the log-rank test. Statistical significance was set at *P* < 0.05. SPSS version 22 (IBM Corp., Armonk, NY, USA) and MedCalc (version 19) were used for all statistical analyses.

## Results

### Patient baseline characteristics

The clinical parameters and efficacy outcomes of ICI treatment are summarized in Table S1. The mean age was 68.24 ± 9.23 years, and the proportion of male patients was 82.8%. All participants were diagnosed with advanced stage III or IV NSCLC. The proportion of former or current smokers was 78.2%, and the major histological types were adenocarcinoma (49.4%) and squamous cell carcinoma (47.1%). A total of 57.5% (50/87) of patients had high PD-L1 expression, and 32.5% (37/20) had no or low expression of PD-L1. Most patients (89.7%) had an ECOG PS score of 0 or 1. Most patients had received at least one previous systemic treatment.

### Clinical outcomes and survival analysis according to plasma GDF15 levels

**Fig. 1:**
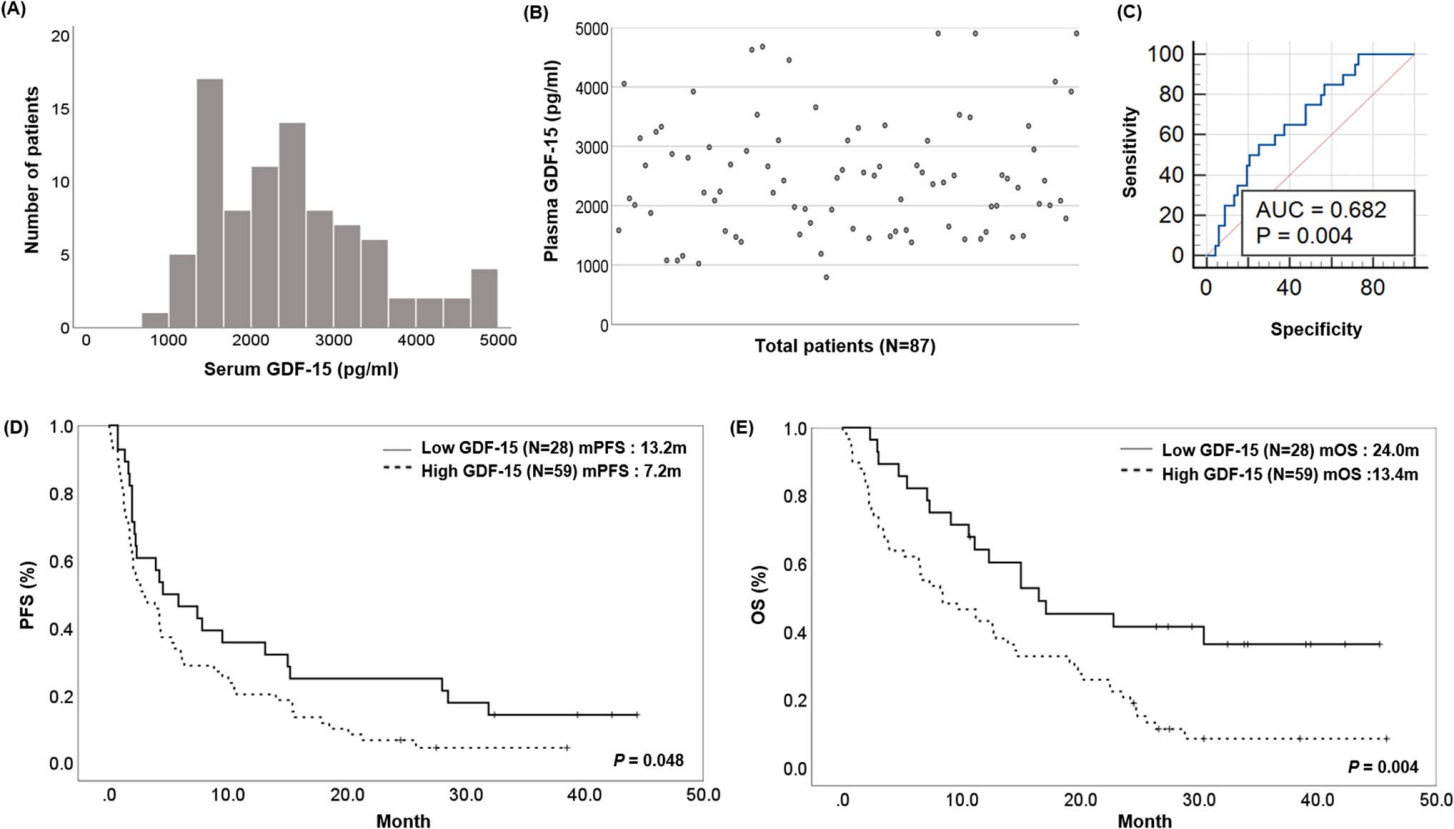
Predictive value and survival analysis according to plasma GDF15 levels in patients with lung cancer treated with PD-1/PD-L1 inhibitors. **A** Histogram of plasma GDF15 levels in all patients. **B** Scatterplot of plasma GDF15 levels in all patients. **C** GDF15 ROC curve for response rate in all patients (*N* = 87). The AUC for GDF15 was 0.682 (*P* = 0.004) based on the 1945 pg/mL cutoff. **D** Progression-free survival (PFS) in total patients (*N* = 87). The median PFS of the low GDF15 group was 13.2 months (95% confidence interval (CI) 7.6–18.9), significantly longer than 7.2 months (95% CI 4.8–9.6, *P* = 0.048) in the high GDF15 group. **E** Overall survival (OS) in total patients (*N* = 87). The median OS of patients in the low GDF15 group (24 months, 95% CI 17.5–30.5) was significantly longer than the OS in the high GDF15 group (13.4 months, 95% CI 10–16.8, *P* = 0.004)

The distribution of baseline plasma GDF15 levels among patients is shown in Fig. 1A, B. The median of plasma GDF15 levels was 2482.47 pg/mL. To investigate the predictive role of GDF15 in patients who underwent immunotherapy, we first identified the ROC curve to distinguish patients with responses from the total population (Fig. 1C). The AUC for GDF15 was 0.682 (*P* = 0.004) based on the 1945 pg/mL cutoff. This cutoff value was higher than the result of 1465 pg/mL of GDF15 threshold associated with recurrence reported in a previous study focused on patients with early NSCLC (stage I–II) and supports the association of GDF15 with advanced disease (Liu et al. 2016).

Based on the cutoff value for baseline GDF15 determined by ROC curve analysis (1945 pg/mL), all patients were classified into the low GDF15 group (28 patients) or high GDF15 group (59 patients). Although the baseline clinical parameters, including age, sex, PD-L1 expression, histological type, type of agents, ECOG, clinical stage, and number of metastatic sites, were not significantly different between the two groups, the objective response rate (ORR) was significantly higher in the low GDF15 group than in the high GDF15 group (39.2% vs. 15.3%, *P* = 0.013). DCR was also significantly higher in the low GDF15 group than in the high GDF15 group (67.8% vs. 45.8%, *P* = 0.044) (Table 1). We performed univariate and multivariate analysis for factors, including GDF15 levels, ECOG performance status, and PD-L1 expression, associated with the response rate to PD-1/PD-L1 inhibitors. In univariate analysis, patients who responded to PD-1/PD-L1 inhibitors were positively associated with better performance status, high PD-L1 expression, and low GDF15 levels than non-responders. Multivariate logistic regression analyses showed that only GDF15 level was significantly associated with response to immunotherapy (Table S2). The median PFS of the low GDF15 group was 13.2 months (95% confidence interval (CI) 7.6–18.9), significantly longer than 7.2 months (95% CI 4.8–9.6, *P* = 0.048) in the high GDF15 group (Fig. 1D). The median OS of patients in the low GDF15 group (24 months, 95% CI 17.5–30.5) was significantly longer than the OS in the high GDF15 group (13.4 months, 95% CI 10–16.8, *P* = 0.004; Fig. 1E). These results are based on a data cutoff from June 2, 2022.

**Table 1:** Comparison of clinical parameters and response according to plasma GDF15 levels in total patients (*N* = 87)

Variable	Low GDF15 group (*N* = 28)	High GDF15 group (*N* = 59)	*P* value
Age, years	66.2 ± 7.5	69.2 ± 9.9	0.123
Sex			
Male	23 (82.1)	49 (83.1)	0.917
Female	5 (17.9)	10 (16.9)	
Smoking status			
Never	8 (28.6)	11 (18.6)	0.295
Former/current	20 (71.4)	48 (81.4)	
ECOG			
0	7 (25.0)	5 (8.5)	0.104
1	19 (67.9)	47 (79.7)	
2	2 (7.1)	7 (11.9)	
Histology			
Adenocarcinoma	14 (50.0)	27 (45.8)	0.473
Squamous	14 (50.0)	29 (49.2)	
Other^a^	0 (0.0)	3 (5.1)	
Stage			
IIIA	1 (3.6)	3 (5.1)	0.993
IIIB	4 (14.3)	7 (11.9)	
IIIC	1 (3.6)	2 (3.4)	
IVA	10 (35.7)	20 (33.9)	
IVB	12 (42.9)	27 (45.8)	
Metastatic sites	1.2 ± 1.1	1.4 ± 1.1	0.414
PD-L1 expression^b^			
No (TPS < 1%)	7 (25.0)	14 (23.7)	0.792
Low (TPS 1–49%)	4 (14.3)	12 (20,3)	
High (TPS ≥ 50%)	17 (60.7)	33 (55.9)	
PD-L1 expression (mean ± SD, %)	37.4 ± 32.3	35.9 ± 20.5	0.833
Number of prior regimens			
0	5 (17.9)	6 (10.2)	0.329
1	21 (75.0)	43 (72.9)	
≥ 2	2 (7.1)	10 (16.9)	
Agent			
Nivolumab	9 (32.1)	17 (28.8)	0.695
Pembrolizumab	4 (14.3)	13 (22.0)	
Atezolizumab	15 (53.6)	29 (49.2)	
Response			
PR	11 (39.2)	9 (15.3)	0.034
SD	8 (28.6)	18 (30.5)	
PD	9 (32.1)	32 (54.2)	
ORR	39.2%	15.3%	0.013
DCR	67.8%	45.8%	0.044
Treatment duration of ICI (mean, months)	6.68	4.39	0.040

Data are presented as mean ± standard deviation (SD) or number of patients (%)

*PD-L1* programmed death ligand 1, *TPS* tumor proportion score, *PR* partial response, *SD* stable disease, *PD* progressive disease, *ORR* objective response rate, *DCR* disease control rate

^a^Two large cells, one non-small cell lung cancer not otherwise specified

^b^Classification of subgroups according to PD-L1 expression was based on the results of the 22C3 pharmDx assay, and patients without 22C3 pharmDx assay results were classified based on the SP263 assay

Collectively, plasma GDF15 levels were significantly associated with the efficacy and prognosis of immunotherapy in patients with advanced NSCLC.

### Comparison of immune cell populations, sPD-1, and sPD-L1 between the low GDF15 group and high GDF15 groups in patients with lung cancer

A total of 54 patients underwent flow cytometry analysis by collecting PBMCs. Since we previously reported that circulating regulatory T (Treg) cells represent a promising potential dynamic biomarker to predict efficacy after immunotherapy in patients with NSCLC (Kang et al. 2022), we analyzed the immune cell populations, including Treg cells, soluble PD-1, and soluble PD-L1 levels between the low GDF15 group and high GDF15 group in patients with lung cancer (Table 2).

**Table 2:** Comparison of immune cell types between the low GDF15 and high GDF15 groups in patients with lung cancer who underwent peripheral blood mononuclear cell (PMBC) flow cytometric analysis (*N* = 54)

Variable	Low GDF15 group (*N* = 13)	High GDF15 group (*N* = 41)	*P* value
CD3^+^	34.3 ± 11.6	28.8 ± 11.4	0.134
CD4^+^	20.7 ± 7.6	15.8 ± 6.8	0.052
CD8^+^	11.2 ± 4.3	10.8 ± 5.9	0.832
PD1^+^CD4^+^	21.7 ± 7.0	22.7 ± 11.3	0.785
PD1^+^CD8^+^	42.1 ± 26.4	24.3 ± 11.9	0.034
Treg/CD4^+^	4.5 ± 1.3	4.7 ± 1.8	0.742
PD1^+^/Treg	7.7 ± 3.9	12.2 ± 6.4	0.019
PD1^+^Treg/CD4^+^	0.47 ± 0.31	0.84 ± 0.64	0.006
Naive Treg [Fraction (Fr.) I]	1.00 ± 0.51	1.08 ± 0.68	0.675
Effector Treg (Fr. II)	0.94 ± 0.61	0.91 ± 0.71	0.867
Non-Treg cells (Fr. III)	3.78 ± 1.18	3.57 ± 1.35	0.612
Soluble PD-1	460.1 ± 808.2	534.8 ± 438.0	0.696
Soluble PD-L1	47.6 ± 27.0	46.9 ± 29.8	0.944

Data are given as mean ± standard deviation (SD)

*P* value from unpaired *t *test for continuous parametric variables and Mann–Whitney *U* test for nonparametric variables

**Fig. 2:**
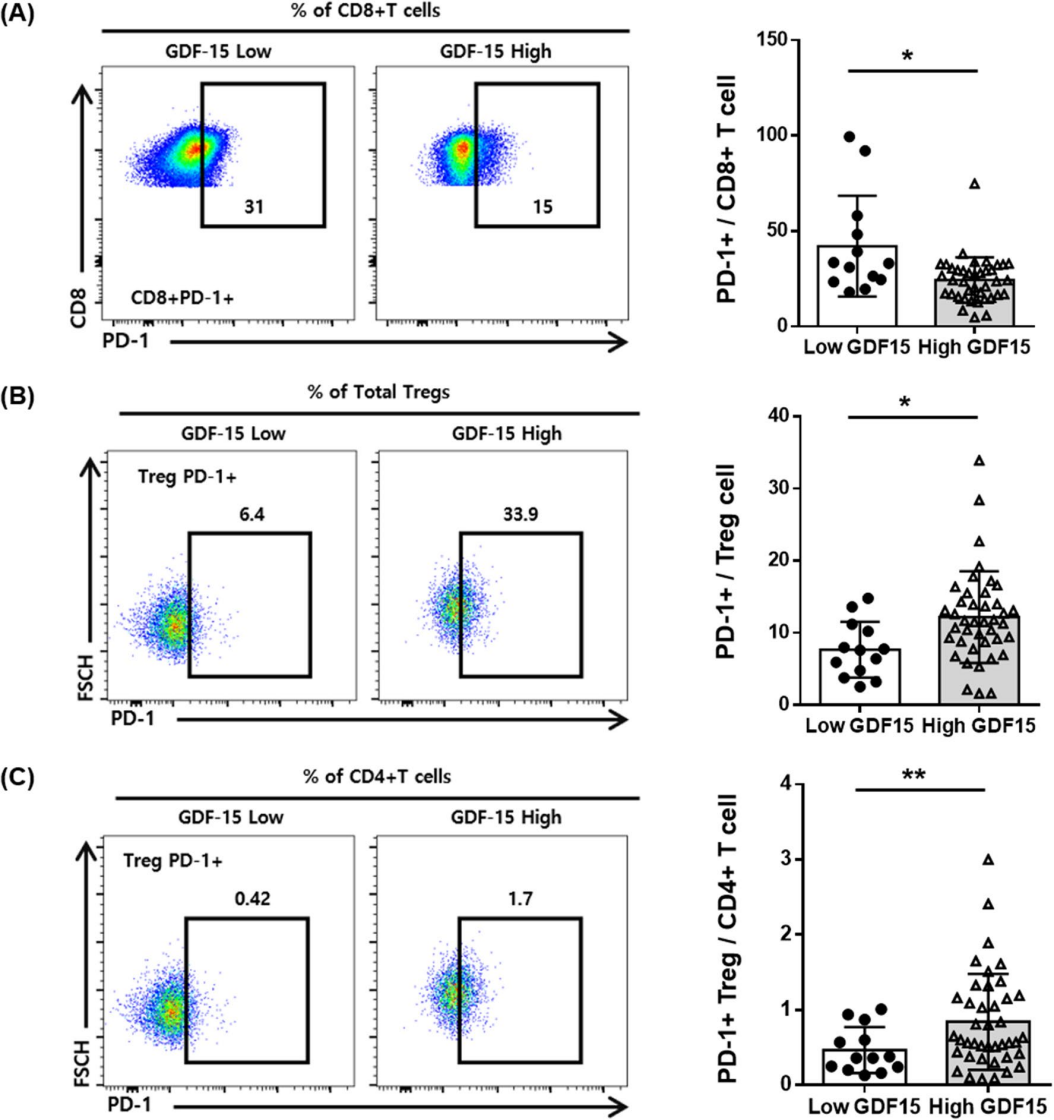
T cell subsets according to plasma GDF15 levels in patients with lung cancer treated with PD-1/PD-L1 inhibitors. **A** The frequency of PD-1^+^ CD8+ T cells among CD8+ T cells was significantly lower in the high GDF15 group than in the low GDF15 group. **B** The frequency of PD-1^+^ Treg cells in Treg cells was significantly higher in the high GDF15 group than in the low GDF15 group. **C** The frequency of PD-1^+^ Treg cells among CD4+ T cells was significantly higher in the high GDF15 group than in the low GDF15 group

The gating strategies applied in the flow cytometric analyses are shown in Fig. S1. Representative images of T cell subsets according to plasma GDF15 levels are shown in Fig. 2. There were no significant differences in CD3^+^, CD4^+^, CD8^+^ T cells, and Treg cells between the two groups (Table 2). Interestingly, the proportion of PD-1^+^ CD8^+^ T cells among CD8^+^ T cells (PD-1^+^/CD8^+^) was significantly lower in the high GDF15 group than in the low GDF15 group (*P* = 0.034, Table 2, Fig. 2A). Furthermore, the proportion of PD-1^+^ Treg cells among Treg cells (PD-1^+^/Treg) and the proportion of PD-1^+^ Treg cells among CD4^+^ T cells (PD-1^+^ Treg/CD4^+^) were significantly higher in the high GDF15 group than in the low GDF15 group (Table 2, Fig. 2B, C). There was no significant difference in soluble PD-1 and PD-L1 levels between the two groups. Since PD-L1 expression in tumor tissues, such as soluble PD-1 and PD-L1, was also not significantly related to plasma GDF15 levels, these results suggest an important association between PD-1^+^ CD8^+^ T cells and PD-1^+^ Treg cells and plasma GDF15 levels in advanced NSCLC patients treated with PD-1/PD-L1 inhibitors.

### Correlation analysis of plasma GDF15 levels with immune cell populations, sPD-1, and sPD-L1 in patients with advanced NSCLC who underwent immunotherapy

**Fig. 3:**
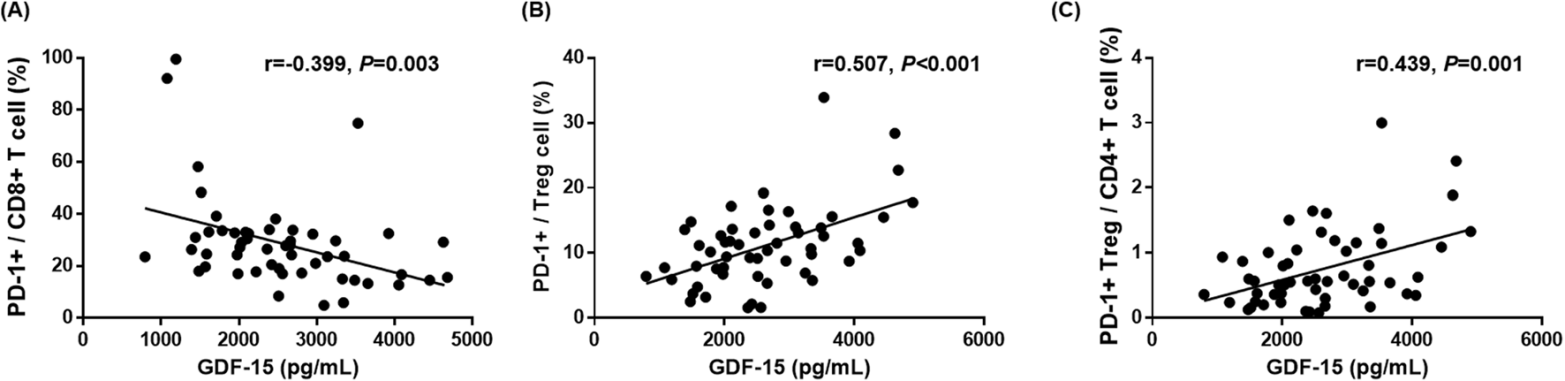
Correlation plots of GDF15 levels with T cell subsets. **A** Plasma GDF15 level were negatively correlated with PD-1+/CD8+ T cells (*r* = − 0.399, *P* = 0.003). **B** Plasma GDF15 level were positively correlated with PD-1+/Treg cells (*r* = 0.507, *P* < 0.001). **C** Plasma GDF15 levels were positively correlated with PD-1+Treg/CD4+ T cells (*r* = 0.439, *P* < 0.001)

To assess the precise relationship between plasma GDF15 levels and immune cell populations, we performed a correlation analysis of plasma GDF15 levels with immune cell sub-populations, sPD-1, and sPD-L1 (Table S3). This showed that plasma GDF15 levels were negatively correlated with PD-1^+^/CD8^+^ (*r* = − 0.399, *P* = 0.003) and positively correlated with PD-1^+^/Treg (*r* = 0.507, *P* < 0.001) and PD-1^+^ Treg/CD4^+^ (*r* = 0.439, *P* < 0.001) (Table S3, Fig. 3). Collectively, plasma GDF15 levels were associated with the proportion of PD-1^+^CD8^+^ T cells and PD-1^+^ Treg cells in patients with advanced NSCLC who underwent immunotherapy.

### Relationship between changes in plasma GDF15 levels during treatment with efficacy and prognosis in advanced NSCLC patients undergoing immunotherapy

**Fig. 4:**
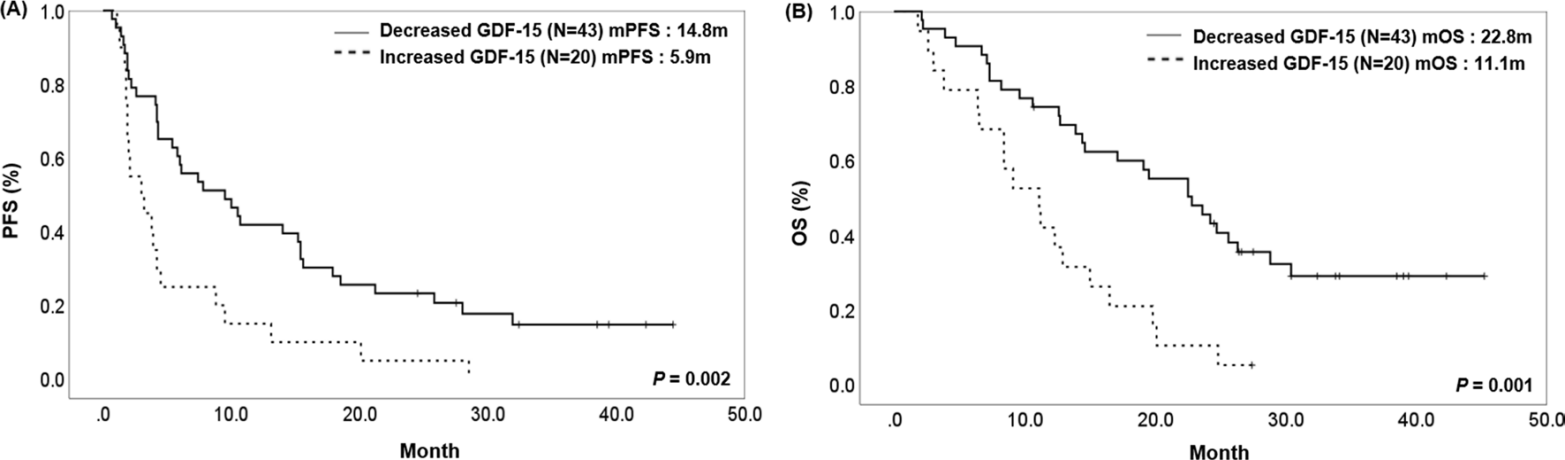
Survival analysis according to change of plasma GDF15 levels in patients with lung cancer treated with PD-1/PD-L1 inhibitors. **A** Progression-free survival (PFS). The median PFS in the decreased GDF15 levels group was 14.8 months (95% CI 10.4–19.2), significantly longer than 5.9 months (95% CI 2.8–9, *P* = 0.002). **B** Overall survival (OS). The median OS in the decreased GDF15 levels group was 22.8 months (95% CI 16.9–28.7), significantly longer than 11.1 months (95% CI 7.1–15, *P* = 0.001)

We divided the groups according to whether GDF15 levels increased or decreased at the first response evaluation after the administration of the PD-1/PD-L1 inhibitor. A total of 63 patients had follow-up blood samples available to measure GDF15 levels. For 2 months, 20 patients were in the increased GDF15 levels group, while 43 patients were in the decreased levels group. There were no significant differences in baseline characteristics and baseline GDF15 levels between the two groups. In the group with decreased GDF15 levels from baseline, the ORR and DCR were significantly higher than in increased levels group (ORR 37.2% vs. 10.0%, *P* = 0.026, DCR 76.7% vs. 40.0%, *P* = 0.004) (Table 3). The median PFS and OS in the decreased GDF15 levels group was 14.8 months (95% CI 10.4–19.2) and 22.8 months (95% CI 16.9–28.7), significantly longer than 5.9 months (95% CI 2.8–9, *P* = 0.002) and 11.1 months (95% CI 7.1–15, *P* = 0.001) (Fig. 4). Our results suggest that changes in plasma GDF15 levels during treatment may serve as predictive and prognostic biomarkers in patients with advanced NSCLC who underwent immunotherapy.

**Table 3:** Baseline characteristics and clinical outcomes according to change of plasma GDF15 levels after immunotherapy in patients with lung cancer with follow-up blood samples after treatment (*N* = 63)

Variable	Decreased GDF15 group (*N* = 43)	Increased GDF15 group (*N* = 20)	*P* value
Age, years	68.7 ± 9.2	70.2 ± 6.2	0.435
Baseline GDF15 levels (pg/ml)	2310.9 ± 822.3	2263.5 ± 731.2	0.826
Sex			
Male	39 (90.7)	16 (80)	0.235
Female	4 (9.3)	4 (20)	
Smoking status			
Never	5 (11.6)	5 (25)	0.176
Former/current	38 (88.4)	15 (75)	
Histology			
Adenocarcinoma	16 (37.2)	11 (55)	0.258
Squamous	16 (55.8)	9 (45)	
Other^a^	3 (6.9)	0 (0)	
PD-L1 expression^b^			
No (TPS < 1%)	9 (20.9)	8 (40)	0.248
Low (TPS 1–49%)	6 (13.9)	3 (15)	
High (TPS ≥ 50%)	28 (65.1)	9 (45)	
Agent			
Nivolumab	6 (13.9)	1 (5)	0.066
Pembrolizumab	26 (60.4)	11 (55)	
Atezolizumab	11 (25.5)	8 (40)	
Response			
PR	16 (37.2)	2 (10)	0.010
SD	17 (39.5)	6 (30)	
PD	10 (23.3)	12 (60)	
ORR	37.2%	10.0%	0.026
DCR	76.7%	40.0%	0.004
Treatment duration of ICI (mean, months)	6.5	3.5	0.052

*PD-L1* programmed death ligand 1, *TPS* tumor proportion score, *PR* partial response; *SD* stable disease, *PD* progressive disease, *ORR* objective response rate, *DCR* disease control rate

^a^Two large cells, one non-small cell lung cancer not otherwise specified

^b^Classification of subgroups according to PD-L1 expression was based on the results of the 22C3 pharmDx assay, and patients without 22C3 pharmDx assay results were classified based on the SP263 assay

## Discussion

To our knowledge, this is the first study to demonstrate whether GDF15 can predict the efficacy of PD-1/PD-L1 inhibitors and identify the association between circulating GDF15 levels and immune cell populations of PBMCs in patients with advanced NSCLC. In this study, patients with high baseline plasma GDF15 levels had lower response rates and poorer PFS and OS than those with low baseline plasma GDF15 levels. Plasma GDF15 levels were negatively correlated with the proportion of PD-1^+^ CD8^+^ T cells and significantly positively correlated with the proportion of PD-1^+^ Treg cells. In addition, after 2 months of treatment, the group with increased GDF15 levels had a lower response rate and poorer PFS and OS than those with decreased GDF15 levels.

In large-scale screenings, GDF15 is the most prominently overexpressed soluble factor across a large range of cancer types, including NSCLC (Welsh et al. 2003). For this reason, GDF15 was proposed as a diagnostic biomarker for early-stage lung cancer (Liu et al. 2016), and correlations between GDF15 and progression have been described for gastric (Blanco-Calvo et al. 2014), colorectal (Li et al. 2016), hepatocellular (Liu et al. 2015), NSCLC (Liu et al. 2016), renal cell (Traeger et al. 2019), melanoma (Weide et al. 2016), and oral cancers (Schiegnitz et al. 2012). In studies on various cancers, the plasma/serum threshold of GDF15 was reported to be 1000–1500 pg/mL. In this study, the cutoff value of GDF15 was 1945 pg/mL based on the ROC curve for treatment response, which was higher than the previously reported threshold. Because this study enrolled patients with advanced-stage rather than early-stage disease, it is thought that GDF15 levels in the overall population would be high. These results suggest that GDF15 levels differ for each cancer type and even for the stage of the same cancer. In addition, plasma GDF15 levels of enrolled patients did not correlate with age in this study, meaning that the changes caused by lung cancer are larger than the changes caused by general aging. GDF15 was known to be upregulated in NSCLC tissues compared to paired normal tissues and was tightly correlated with poor clinical outcomes, such as tumor size, lymph node metastasis and TNM stage in NSCLC (Lu et al. 2018; Zhao et al. 2018a, b). Blood biomarkers can be easily measured, and for advanced stages, most small biopsies are performed instead of surgery, with the advantage of usage even when IHC is difficult. Our results suggest that not only the baseline, but also the change in plasma GDF15 level predicts the response of ICIs, which we think is a very interesting and important result. After ICI administration, atypical responses such as pseudo-progression and hyper-progression may occur, and for most patients the choice to perform re-biopsy in a situation to distinguish between progression and an immune-related reaction is a difficult one. In these patients, plasma GDF15 level can be a useful non-invasive biomarker.

Although GDF15 is a well-known poor prognostic factor in a wide variety of cancers, the potential role of circulating GDF15 as a candidate predictor of chemoresistance and clinical outcomes has not yet been investigated comprehensively. In a study of epithelial ovarian cancer, chemo-resistant patients showed significantly higher GDF15 levels than chemo-sensitive patients, and high expression of GDF15 was an independent negative prognostic indicator of PFS (Zhao et al. 2018a, b). This study is different from palliative treatment in the advanced stages of our study, because it targeted patients who received adjuvant platinum-based chemotherapy after surgery. Additionally, evaluation for the value of GDF15 in lung cancer chemotherapeutic response showed that GDF15 levels were significantly decreased in all patients after two cycles of treatment, regardless of the type of response to treatment; the effect was greater in the PR group (Deng et al. 2021). In an abstract presented at the 2020 American Association for Cancer Research, it was reported that GDF15 levels in patients with melanoma were predictive of the clinical response to anti-PD1 treatment (Wischhusen et al. 2020a, b). However, since the entire paper has not been published, the cutoff value of GDF15 levels, treatment response, and survival data cannot be accurately established, and the change in GDF15 levels has not been studied. As a potential biomarker for immunotherapy, GDF15 was included in a study exploring the possible predictors of resistance to anti-CTLA-4 (ipilimumab) therapy. However, GDF15 was not a significant predictor in multivariate analysis (Nyakas et al. 2019). In our study, the plasma GDF15 level of non-responder patients was 2626.99 ± 1025.79 pg/mL, which was significantly higher than the GDF15 level of responder patients at 1998.35 ± 654.09 pg/mL (*P* = 0.012). We confirmed that GDF15 levels can predict the response to ICIs regardless of disease burden, such as disease stage or number of metastatic sites. Additionally, GDF15 was identified as the only significant factor when multivariate analysis was performed with ECOG performance and PD-L1 expression, which were previously known as predictors of ICI response. It is considered that GDF15 has a predictive role for PD-1/PD-L1 inhibitor treatment in patients with lung cancer. Prediction of drug sensitivity before treatment could help clinicians select an appropriate treatment regimen to customize individualized treatment strategies. In addition, substances targeting GDF15 such as human GDF15 blocking peptide and GDF15 antibody have already been developed. Of course, clinical trials are required, but the therapeutic option of administering GDF15 blockade together with PD-1/PD-L1 blockers can be considered in patients with high baseline GDF15 before treatment. In other words, through this study, GDF15 can be considered not only as a biomarker that can predict the response to ICI, but also as a new therapeutic target that overcomes resistance to immunotherapy.

GDF15 is a stress-induced cytokine secreted by tumor cells that is affected by tumor-promoting inflammation and immune infiltration (Zhou et al. 2013). In a previous study on the predictive role of GDF15 in anti-PD1 treatment in patients with melanoma, intratumoral GDF15 levels in melanoma correlated inversely with CD3^+^ and CD8^+^ T cell infiltration (Wischhusen et al. 2020a, b). In murine prostate cancer, GDF15 overexpression was associated with increased CD8^+^ T cell numbers and a reduced proportion of CD8^+^PD-1^+^ T cells (Husaini et al. 2020). In the present study, we found that GDF15 levels and the proportion of PD-1^+^CD8^+^ T cells among CD8^+^ T cells were negatively correlated. Although several studies suggested PD-1^+^ CD8^+^ T cells lead to impaired T cell functions and tumor escape, there are also studies related to their efficient role in immune T cell responses (Simon and Labarriere 2018). Thus, the role of PD-1^+^ CD8^+^ T cells in tumors is still controversial. However, it is now clear that PD-1 expression is first a marker of T cell activation, allowing the identification of the tumor-reactive CD8^+^ T cell fraction in tumors (Inozume et al. 2010, Gros et al. 2014). The proliferation of PD-1^+^ CD8^+^ T cells in the blood after PD-1 blockade treatment has been reported to be associated with positive clinical outcomes (Kamphorst et al. 2017). These results support the possibility of a role in effective immune response of PD-1^+^ CD8^+^ T cells, which showed a negative correlation with GDF15 levels observed in our study. In addition, we found that GDF15 levels were negatively correlated with the proportion of PD-1^+^ Treg cells among Treg cells and CD4^+^ T cells. In hepatocellular carcinoma (HCC) tissues, GDF15 was reported to be positively associated with elevated Treg cell frequency (Wang et al. 2021). However, we investigated circulating GDF15 levels and Treg cell frequency in PBMC, which differs from existing literature. Since GDF15 is not only secreted by tumor cells but also is affected by various immune cells, and immunotherapy targets T cells, it may be more useful to measure GDF15 at the circulating level. Furthermore, GDF15 contributes to regulatory T cell-mediated suppression of conventional T cell activation and inflammatory cytokines (Moon et al. 2020). We previously reported that circulating PD-1^+^ Treg cells were significantly decreased in the responder group of patients with lung cancer treated with PD-1/PD-L1 inhibitors (Kang et al. 2022). It is thought that GDF15 affects the fraction of PD-1^+^ CD8^+^ T cells and PD-1^+^ Treg cells in patients with lung cancer, and this is related to the response to immunotherapy.

This study had some limitations. First, this study was performed in a single center; therefore, the number of patients involved in this study was small. Thus, our results should be validated in larger cohorts. Second, through analysis of the relationship between GDF15 and the circulating immune cell population, we have established some hypotheses about the effect of GDF15 levels on treatment response to ICIs, but the exact basic mechanism has not been fully investigated. Additional experimental studies are needed to determine why the efficacy of immune checkpoint blockade is low when circulating GDF15 levels are elevated.

## Conclusions

In this study, we demonstrated that baseline GDF15 levels and changes in these after treatment correlated with clinical benefits and survival in patients with NSCLC treated with PD-1/PD-L1 inhibitors. We also found that plasma GDF15 levels were associated with the proportion of circulating PD-1^+^CD8^+^ T cells and PD-1^+^ Treg cells. In conclusion, our data suggest that GDF15 could be a potential biomarker for predicting efficacy and survival benefit and may be a new therapeutic target that can overcome resistance to immunotherapy in patients with NSCLC.

## Supplementary Information

Below is the link to the electronic supplementary material.

Supplementary file1 Figure S1 Gating strategies by flow cytometric analyses (TIF 206 KB)

Supplementary file2 (DOCX 24 KB)

## Data Availability

The data presented in this study are available on request from the corresponding author.
