# Supplementary figures and images for: Plasma GDF15 levels associated with circulating immune cells predict the efficacy of PD-1/PD-L1 inhibitor treatment and prognosis in patients with advanced non-small cell lung cancer
Source: J Cancer Res Clin Oncol. 2022 Dec 6;149(1):159–71. doi: 10.1007/s00432-022-04500-5 (PMC9889409; doi:10.1007/s00432-022-04500-5)

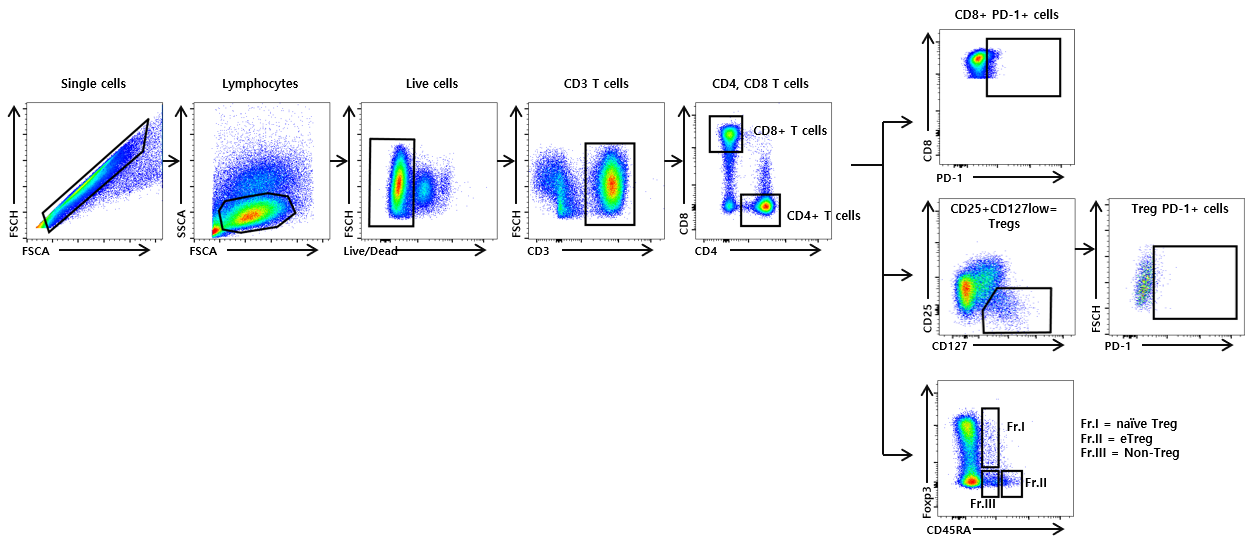

Supplement: Supplementary file 1 — Supplementary file1 Figure S1 Gating strategies by flow cytometric analyses (TIF 206 KB) [file 432_2022_4500_MOESM1_ESM.tif]
