# Supplementary material for: Plasma GDF15 levels associated with circulating immune cells predict the efficacy of PD-1/PD-L1 inhibitor treatment and prognosis in patients with advanced non-small cell lung cancer
Source: J Cancer Res Clin Oncol. 2022 Dec 6;149(1):159–71. doi: 10.1007/s00432-022-04500-5 (PMC9889409; doi:10.1007/s00432-022-04500-5)
Supplement: Supplementary file 2 — Supplementary file2 (DOCX 24 KB) [file 432_2022_4500_MOESM2_ESM.docx]

Supplementary Table 1. Baseline characteristics and efficacy outcomes in all patients (N = 87).

| Variable |  | Mean ± standard deviation or number of patients (%) |
| --- | --- | --- |
| Age, years |  | 68.24 ± 9.23 |
| Sex | Male | 72 (82.8) |
|  | Female | 15 (17.2) |
| Smoking status | Never | 19 (21.8) |
|  | Former/Current | 68 (78.2) |
| ECOG | 0 | 12 (13.8) |
|  | 1 | 66 (75.9) |
|  | 2 | 9 (10.3) |
| Histology | Adenocarcinoma | 43 (49.4) |
|  | Squamous | 41 (47.1) |
|  | Other^*^ | 3 (3.4) |
| Stage | IIIA | 4 (4.6) |
|  | IIIB | 11 (12.6) |
|  | IIIC | 3 (3.4) |
|  | IVA | 30 (34.5) |
|  | IVB | 39 (44.8) |
| EGFR | Mutant | 0 |
| ALK rearrangement | Positive | 1 (1.1) |
| ROS-1 rearrangement | Positive | 1 (1.1) |
| PD-L1 expression† | No (TPS < 1%) | 21 (24.1) |
|  | Low (TPS 1–49%) | 16 (18.4) |
|  | High (TPS ≥ 50%) | 50 (57.5) |
| Number of prior regimens | 0 | 11 (12.6) |
|  | 1 | 64 (73.6) |
|  | ≥ 2 | 12 (13.8) |
| Agent | Nivolumab | 17 (19.5) |
|  | Pembrolizumab | 44 (50.6) |
|  | Atezolizumab | 26 (29.9) |
| Response to treatment | PR | 20 (23.0) |
|  | SD | 26 (29.9) |
|  | PD | 41 (47.1) |

Abbreviations: ECOG, Eastern Cooperative Oncology Group; EGFR, epidermal growth factor receptor; ALK, anaplastic lymphoma kinase; PD-L1, programmed death-ligand 1; TPS, tumor proportion score; PR, partial response; SD, stable disease; PD, progression disease.^*^ Two large cell, one non-small cell lung cancer not otherwise specified.†The classification of subgroups according to PD-L1 expression was based on the results of the 22C3 pharmDx assay, and patients without 22C3 pharmDx assay results were classified based on the SP263 assay.

Supplementary Table 2. Univariate and multivariate analysis of factors associated with the response rate

|  |  | Univariate |  | Multivariate |  |
| --- | --- | --- | --- | --- | --- |
| Variable |  | Odds ratio (95% CI) | *P* value | Odds ratio (95% CI) | *P* value |
| ECOG | 0 | 6.677 (1.831-24.354) | 0.004 | 3.741 (0.936-14.957) | 0.062 |
|  | 1-2 | 1 |  | 1 |  |
| PD-L1 | No/Low | 1 | 0.026 | 1 | 0.089 |
|  | High | 3.882 (1.174-12.835) |  | 3.053 (0.844-11.046) |  |
| GDF15 | Low | 3.595 (1.272-10.157) | 0.016 | 3.095 (1.005-9.535) | 0.049 |
|  | High | 1 |  | 1 |  |

Abbreviations: ECOG, Eastern Cooperative Oncology Group; PD-L1, programmed death-ligand 1; GDF15, Growth differentiation factor-15.

Supplementary table 3. Correlation analysis of serum GDF15 levels with other variables, such as immune cell populations, soluble PD-1, and soluble PD-L1 in lung cancer patients underwent peripheral blood mononuclear cell (PBMC) flow cytometric analysis (N=54).

|  | GDF15 | CD3^+^ | CD4^+^ | CD8^+^ | PD1^+^/CD4^+^ | PD1^+^/CD8^+^ | Treg  /CD4^+^ | PD1^+^/Treg | PD1^+^Treg  /CD4 | naive Treg  (Fr. I) | Effector Treg  (Fr. II) | Non-Treg cells (Fr. III) | sPD-1 | sPD-L1 |
| --- | --- | --- | --- | --- | --- | --- | --- | --- | --- | --- | --- | --- | --- | --- |
| GDF15 |  | -0.145  (0.305) | -220  (0.117) | 0.002  (0.988) | 0.113  (0.425) | -0.399**  (0.003) | -0.063  (0.650) | 0.507**  (<0.001) | 0.439**  (<0.001) | -0.015  (0.913) | 0.053  (0.706) | -0.180  (0.193) | -0.120  (0.434) | 0.211  (0.165) |
| CD3^+^ | -0.145  (0.305) |  | 0.869**  (<0.001) | 0.812**  (<0.001) | -0.274*  (0.049) | 0.042  (0.767) | -0.144  (0.318) | -0.194  (0.177) | -0.221  (0.124) | -0.035  (0.810) | -0.104  (0.474) | -0.239  (0.095) | -0.134  (0.451) | -0.185  (0.295) |
| CD4^+^ | -0.220  (0.117) | 0.869**  (<0.001) |  | 0.435**  (0.001) | -0.357**  (0.009) | 0.077  (0.589) | -0.124  (0.390) | -0.207  (0.150) | -0.270  (0.058) | -0.001  (0.993) | -0.095  (0.511) | -0.183  (0.202) | -0.082  (0.643) | -0.037  (0.837) |
| CD8^+^ | 0.002  (0.988) | 0.812**  (<0.001) | 0.435**  (0.001) |  | -0.131  (0.356) | -0.008  (0.955) | -0.106  (0.465) | -0.147  (0.308) | -0.124  (0.389) | -0.054  (0.709) | -0.045  (0.756) | -0.203  (0.158) | -0.150  (0.397) | -0.260  (0.137) |
| PD1^+^/CD4^+^ | 0.113  (0.425) | -0.274*  (0.049) | -0.357**  (0.009) | -0.131  (0.356) |  | 0.137  (0.334) | 0.207  (0.149) | 0.340*  (0.016) | 0.426**  (0.002) | -0.189  (0.189) | 0.308*  (0.030) | 0.289*  (0.042) | 0.196  (0.268) | -0.067  (0.706) |
| PD1^+^/CD8^+^ | -0.399**  (0.003) | 0.042  (0.767) | 0.077  (0.589) | -0.008  (0.955) | 0.137  (0.334) |  | 0.073  (0.616) | -0.191  (0.185) | -0.115  (0.425) | -0.027  (0.853) | -0.014  (0.924) | 0.102  (0.480) | 0.061  (0.732) | -0.023  (0.896) |
| Treg/CD4^+^ | -0.063  (0.650) | -0.144  (0.318) | -0.124  (0.390) | -0.106  (0.465) | 0.207  (0.149) | 0.073  (0.616) |  | 0.048  (0.732) | 0.386**  (0.004) | 0.439**  (0.001) | 0.752**  (<0.001) | 0.778**  (<0.001) | -0.016  (0.924) | 0.037  (0.826) |
| PD1^+^/Treg | 0.507**  (<0.001) | -0.194  (0.177) | -0.207  (0.150) | -0.147  (0.308) | 0.340*  (0.016) | -0.191  (0.185) | 0.048  (0.732) |  | 0.876**  (<0.001) | 0.038  (0.785) | 0.158  (0.253) | -0.152  (0.272) | -0.036  (0.832) | 0.021  (0.904) |
| PD1^+^Treg  /CD4^+^ | 0.439**  (0.001) | -0.221  (0.124) | -0.270  (0.058) | -0.124  (0.389) | 0.426**  (0.002) | -0.115  (0.425) | 0.386**  (0.004) | 0.876**  (<0.001) |  | 0.120  (0.386) | 0.400**  (0.003) | 0.129  (0.352) | 0.054  (0.750) | 0.117  (0.490) |
| naive Treg  (Fraction (Fr.). I) | -0.015  (0.913) | -0.035  (0.810) | -0.001  (0.993) | -0.054  (0.709) | -0.189  (0.189) | -0.027  (0.853) | 0.439**  (0.001) | 0.038  (0.785) | 0.120  (0.386) |  | 0.250  (0.068) | 0.0233  (0.869) | 0.097  (0.568) | -0.018  (0.914) |
| Effector Treg  (Fr. II) | 0.053  (0.706) | -0.104  (0.474) | -0.095  (0.511) | -0.045  (0.756) | 0.308**  (0.030) | -0.014  (0.924) | 0.752**  (<0.001) | 0.158  (0.253) | 0.400**  (0.003) | 0.250  (0.068) |  | 0.575**  (<0.001) | -0.073  (0.668) | 0.046  (0.787) |
| Non-Treg cells (Fr. III) | -0.180  (0.193) | -0.239  (0.095) | -0.183  (0.202) | -0.203  (0.158) | 0.289*  (0.042) | 0.102  (0.480) | 0.778**  (<0.001) | -0.152  (0.272) | 0.129  (0.352) | 0.023  (0.869) | 0.575**  (<0.001) |  | -0.120  (0.480) | -0.158  (0.349) |
| sPD-1 | -0.120  (0.434) | -0.134  (0.451) | -0.082  (0.643) | -0.150  (0.397) | 0.196  (0.268) | 0.061  (0.732) | -0.016  (0.924) | -0.036  (0.832) | 0.054  (0.750) | 0.097  (0.568) | -0.073  (0.668) | -0.120  (0.480) |  | 0.225  (0.137) |
| sPD-L1 | 0.211  (0.165) | -0.185  (0.295) | -0.037  (0.837) | -0.262  (0.137) | -0.067  (0.706) | -0.023  (0.896) | 0.037  (0.826) | 0.021  (0.904) | 0.117  (0.490) | -0.018  (0.914) | 0.046  (0.787) | -0.158  (0.349) | 0.224  (0.137) |  |

^a^ Coefficients (r) were calculated using Pearson’s method. * *P* value <0.05. ** *P* value <0.01.
